# Electrospun Membranes Based on Polycaprolactone, Nano-Hydroxyapatite and Metronidazole

**DOI:** 10.3390/ma14040931

**Published:** 2021-02-16

**Authors:** Ioana-Codruţa Mirică, Gabriel Furtos, Ondine Lucaciu, Petru Pascuta, Mihaela Vlassa, Mărioara Moldovan, Radu-Septimiu Campian

**Affiliations:** 1 Department of Oral Health, Iuliu Hatieganu University of Medicine and Pharmacy, Victor Babes Street 15, 400012 Cluj-Napoca, Romania; mirica_codruta@yahoo.com (I.-C.M.); ondineluc@yahoo.com (O.L.); rcampian@email.com (R.-S.C.); 2 Department of Dental Materials, Babes-Bolyai University-Raluca Ripan, Institute of Research in Chemistry, Fantanele Street 30, 400294 Cluj-Napoca, Romania; mihaela_cecilia@yahoo.com (M.V.); mmarioara2004@yahoo.com (M.M.); 3 Technical University of Cluj-Napoca, Memorandumului Street 28, 400114 Cluj-Napoca, Romania; petru.pascuta@phys.utcluj.ro

**Keywords:** metronidazole, bone regeneration, mechanical properties, biomaterial, electrospinning

## Abstract

The aim of this research was to develop new electrospun membranes (EMs) based on polycaprolactone (PCL) with or without metronidazole (MET)/nano-hydroxyapatite (nHAP) content. New nHAP with a mean diameter of 34 nm in length was synthesized. X-ray diffraction (XRD) and attenuated total reflectance Fourier transform infrared spectroscopy (FTIR-ATR) were used for structural characterization of precursors and EMs. The highest mechanical properties (the force at maximum load, Young’s modulus and tensile strength) were found for the PCL membranes, and these properties decreased for the other samples in the following order: 95% PCL + 5% nHAP > 80% PCL + 20% MET > 75% PCL + 5% nHAP + 20% MET. The stiffness increased with the addition of 5 wt.% nHAP. The SEM images of EMs showed randomly oriented bead-free fibers that generated a porous structure with interconnected macropores. The fiber diameter showed values between 2 and 16 µm. The fiber diameter increased with the addition of nHAP filler and decreased when MET was added. New EMs with nHAP and MET could be promising materials for guided bone regeneration or tissue engineering.

## 1. Introduction

The evolution of medicine nowadays requires a change in the treatment methods and biomaterials used. Certainly, there is a requirement for biomaterials that can demonstrate complex functions and allow a proper host–material interaction. This shift leads to the development of a new generation of biomaterials [1], characterized by nanofibers with the diameter situated in a submicron range which forms a microporous, tortuous network [2], obtained through the electrospinning process [3,4]. Furthermore, their design makes them appropriate for a variety of medical applications like tissue engineering, because it provides mechanical properties similar to the replaced tissue [5,6]. Drug delivery can also be one of their medical applications [2], which makes them suitable for the treatment of periodontal disease. This condition affects around 36.8% of the adults in the United States [7] and is difficult to treat because it involves Gram-negative, anaerobic microflora [8]. The periodontal disease is usually diagnosed by correlating the clinical examination with the radiological one [9] and can be sustained by measuring different markers of the gingival tissue [10]. The presence of this microflora in the body can have repercussions at different levels. Recently, some studies have reported an association between certain systemic inflammatory diseases, like rheumatoid arthritis or diabetes, and cardiovascular diseases such as coronary heart disease, stroke and endothelial dysfunction [11]. Today’s applied treatment involves two parts, i.e., the surgical part, which can involve bone graft, and the medical part [12], but other biomaterials like the hyaluronic acid are also taken into consideration [13].

The common administration of antibacterial agents like mouthwashes shows poor results in disease control because a small amount of the drugs reach the periodontal pocket and the drugs are constantly flushed from the periodontal pocket, due to a very high fluid-clearance rate (40 replacements of the fluid/hour in a 5 mm pocket) [14]. Clinicians use metronidazole (MET) in the treatment of anaerobic infections. MET requires long periods of administration to be efficient and usually shows side effects like nausea, loss of appetite and metallic taste [15]. Hydroxyapatite (HAP) is similar to natural bone and has shown its use over time for bone grafting [16] and as fillings in periodontal pockets [17]. Furthermore, the use of nano-hydroxyapatite (nHAP) has the advantages of better wettability, high surface area and ability to form of a strong layer with the enamel [17]. The incorporation of HAP in membranes based on polycaprolactone (PCL) showed *in vitro* bioactivity [18]. PCL brings the advantage of being biodegradable and not producing a local acid environment; it is nontoxic and is completely excreted from the human body [19]. The feasibility of an experimentally developed biomaterial can be validated by testing its mechanical properties [20]. Using the above-mentioned specific advantages of precursors PCL, MET and nHAP, we developed new electrospun membranes (EMs).

The aim and novelty of this research was the synthesis of new nHAP using two dispersing agents and the use of the new nHAP for the development of a new EM based on PCL with or without MET/nHAP content. The new nHAP was characterized by transmission electron microscopy (TEM), X-ray diffraction (XRD) and attenuated total reflectance Fourier transform infrared spectroscopy (FTIR-ATR). All precursors and EMs were structurally characterized by XRD and FTIR-ATR. For mechanical characterization, the force at maximum load, Young’s modulus, the stiffness and the tensile strength of EMs were investigated. SEM investigation was used to analyze the topography of EMs.

The null hypotheses tested in the article were as follows: (1) the composition does not affect the structure and the topography of new EMs; (2) the composition does not influence the mechanical properties of EMs.

## 2. Materials and Methods

### 2.1. Materials

PCL with molecular weight (M.W.) 80,000 g·mol^−1^, MET, dichloromethane (DCM), dimethylformamide (DMF), poly(vinyl alcohol) (PVA), diammonium hydrogen phosphate ((NH_4_)_2_HPO_4_), calcium nitrate tetrahydrate (Ca(NO_3_)_2_·4H_2_O) and ammonium hydroxide solution (NH_4_OH) were acquired from Sigma-Aldrich GmbH, Steinheim, Germany. Darvan 821A was purchased from R. T. Vanderbilt, Norwalk, CT, USA. Darvan 821A is an ammonium polyacrylate mainly used as a dispersing agent to avoid the formation of particle aggregates of nHAP in homogeneous and highly concentrated nHAP colloidal suspension. Deionized water was used in all experiments. The resistivity of absolute pure water was 18.2 MΩ·cm at 25 °C or electrical conductivity 0.055 µS/cm. All commercial materials were used without further purification.

### 2.2. Synthesis and Characterization of nHAP

nHAP was synthesized using a wet chemical method from calcium nitrate and ammonium hydrogen phosphate from Ca and P precursors. Two solutions of 1200 mL were prepared in two beaker glasses (3000 mL) by separately mixing calcium nitrate and ammonium hydrogen phosphate with double distilled water by strong stirring in an aqueous solution at ambient temperature. A solution dispersing agent of 0.2 vol.% of 1:1 Darvan 821A/PVA was added to both solutions. Each aqueous solution had the pH adjusted to 10.5 by using 25% NH_4_OH solution. Calcium nitrate solution was mixed with ammonium hydrogen phosphate solution in conformity with the standard stoichiometry for pure HAP at a Ca/P ratio of 1.67. Moreover, nHAP was prepared according to the following reaction:

10Ca(NO_3_)_2_·4H_2_O + 6(NH_4_)_2_HPO_4_ + 8NH_4_OH → Ca_10_(PO_4_)_6_(OH)_2_ + 20NH_4_NO_3_ + 20H_2_O (1)

The solution of (NH_4_)_2_HPO_4_ was added dropwise into Ca(NO_3_)_2_ solution at 70 °C with strong stirring, and a pH of 10.5 was maintained by using 25% NH_4_OH solution. The suspension was observed under stirring for 12 h. Then, the formed precipitate was filtrated and washed three times with double distilled water and anhydrous ethanol. Finally, the nHAP particles were lyophilized (Christ alpha 1-4LD Plus model) and dried in an oven at 80 °C for 12 h. After that, the powder was crushed and kept at 300 °C in a furnace for 6 h to obtain a fine nHAP dry powder.

### 2.3. Preparation of Experimental EMs

Four EMs with different compositions were designed and obtained in this study (Table 1). For all the experimentally obtained EMs, PCL was used in the same quantity (2 g), in order to obtain similar thickness for all EMs. nHAP and MET were added in different weight percentages according to Table 1, and weight percentage was calculated with respect to 2 g PCL. PCL and MET were dissolved together in a solvent mixture of DCM/DMF (3:1) and stirred for 3 h at room temperature. After this period, nHAP was added and the stirring continued for another 9 h at room temperature. All bottles (25 mL) containing the electrospinning solutions were sonicated in an ultrasonic bath at 40 kHz for 30 min. To obtain the new EMs, we used a handmade electrospinning machine from Raluca Ripan, Institute of Research in Chemistry, Cluj-Napoca, Romania. The experimental EMs were obtained using a voltage between 12 and 17 kV, a needle tip with 22 G and a flow rate of 2.5 mL·h^−1^. The fibers were collected on a surface of aluminum foil located 23 cm away from the needle tip. During/after the electrospinning process, the solvent evaporated, and the obtained EMs had the composition described in Table 1 and were dried under vacuum for 48 h in a desiccator.

### 2.4. Attenuated Total Reflectance Fourier Transform Infrared Spectroscopy (FTIR-ATR)

To characterize the individual EMs, Fourier transform infrared (FTIR) spectroscopy was carried out in an attenuated total reflection mode (ATR). The spectra were recorded on FTIR spectrophotometer (FTIR-610, Jasco International Co., LTD., Tokyo, Japan) equipped with an ATR attachment with a horizontal ZnSe crystal. The spectra were scanned in the mid-IR range from 400 to 4000 cm^−1^. The resolution of the spectra was 4 cm^−1^, and the scans were repeated 100 times. The spectra were corrected against the background spectrum.

### 2.5. X-ray Diffraction

The XRD measurements were run at room temperature using a Shimadzu 6000 XRD diffractometer (Shimadzu Corporation, Kyoto, Japan), with a monochromator of graphite for the Cu-Kα radiation. The source power was operated at a voltage of 40 kV and current 30 mA, and the scan speed was 2°/min. The PDF2 reference library was used for qualitative identification of the crystalline phases. The average of the mean crystallite size of the synthesized nHAP was determined by using the Debye–Scherrer formula given by Equation (2) [21]:

(2) $$D=\frac{\lambda \cdot K}{\beta \cdot \cos\theta }$$

where D is the apparent volume-weighted crystallite size, λ is the X-ray wavelength (Cu, Kα), K = 0.89 (the Scherrer constant), β is the full width at the half maximum of the (002) line and θ is the angle of Bragg diffraction corresponding to (002) Miller plane.

### 2.6. Transmission Electron Microscopy (TEM) and Scanning Electron Microscopy (SEM)

TEM (H-7650 120 kV automatic microscope, Hitachi, Japan) was used at 80 kV high voltage and 20× magnification to investigate the size and morphology of nHAP. Morphology of the EMs was investigated by scanning electron microscopy (SEM Inspect S, FEI, Eindhoven, Netherlands) using high vacuum, 15 kW, 1000× magnification and 10.7–13.9 mm working distance. The diameter of nHAP from the TEM image and the fiber diameter from SEM image were evaluated using Image J software (U.S. National Institutes of Health, Bethesda, MD, USA). After collecting the data, the mean diameter and standard deviation (SD) were calculated and the graphs were generated.

### 2.7. Mechanical Properties

The developed EMs with a rectangular shape (width = 6 mm, length = 8 mm) were tested for tensile strength (*n* = 5) under dry conditions using a mechanical testing machine (LR5K Plus, Lloyd Instruments, Ltd., Bognor Regis, UK) using a loading rate of 1 mm/min^−1^. The force at maximum load, the Young’s modulus, the stiffness and the tensile strength were evaluated. The Young’s modulus of the samples was measured from the slope of the elastic portion (linear) of the stress–strain curve. The stiffness (**S**) measures the resistance offered by an elastic body to deformation and was calculated using Equation (3):

(3) $$S\;=\;\frac{F}{\delta }\;(N/m)$$

where **F** is the force on the body and **δ** is the displacement produced by the force along the same degree of freedom.

The tensile strength (**TS**) was measured using Equation (4):

(4) $$\mathrm{TS}\;=\;\frac{F}{A}\;(\mathrm{MPa})$$

where **TS** is the tensile strength (MPa), **F** is the force on the cross-section of the sample at final tension (N) and **A** is the nominal cross-sectional area of the sample (mm^2^).

### 2.8. Statistical Analyses

The obtained values were statistically analyzed using the SPSS software package (Version 11.5, SPSS Inc., Chicago, IL, USA) by one-way analysis of variance (ANOVA) and by Tukey’s test with the level of significance set at 0.05 in order to determine the significant differences between the mean values of the tested EMs.

## 3. Results and Discussion

**Figure 1:**
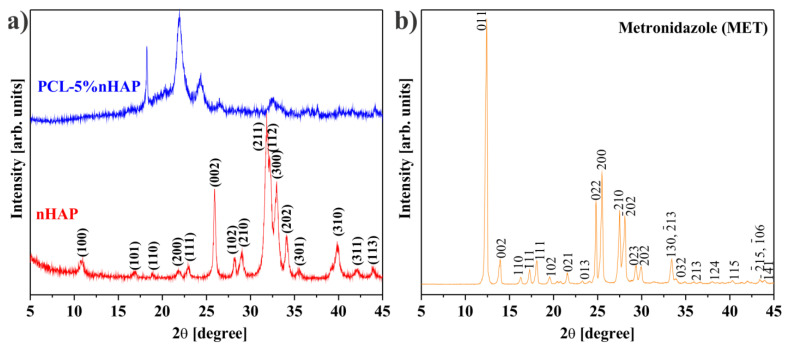
XRD patterns for (**a**) nano-hydroxyapatite (nHAP) and (**b**) metronidazole (MET).

**Figure 2:**
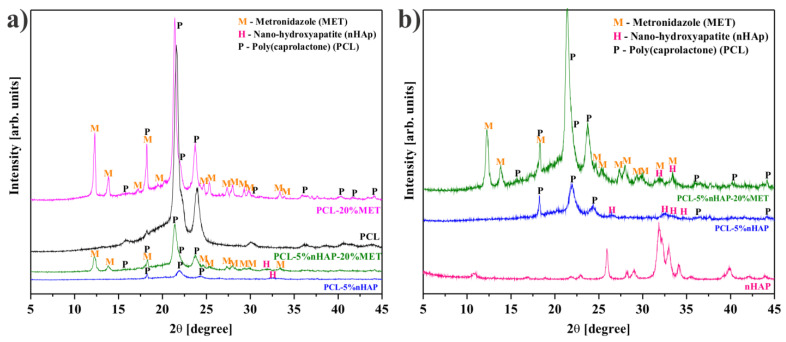
XRD patterns: (**a**) polycaprolactone (PCL), PCL-5% nHAP, PCL-20% MET and PCL-5% nHAP-20% MET; (**b**) nHAP synthesized, PCL-5% nHAP and PCL-5% nHAP-20% MET (to better highlight the presence of nHAP).

The average crystallite of nHAP from XRD measurement (Figure 1a) showed a size of 35.3 nm. All precursors used in the synthesis of the EMs, i.e., PCL, nHAP and MET, and the obtained EMs, i.e., PCL, PCL-5% nHAP, PCL-20% MET and PCL-5% nHAP-20% MET, were analyzed by X-ray diffraction. XRD patterns of PCL (Figure 1 and Figure 2), nHAP (PDF#090432) (Figure 1a) and MET (PDF#381556) (Figure 1b) showed crystalline phases. The obtained EMs, namely PCL, PCL-5% nHAP, PCL-20% MET and PCL-5% nHAP-20% MET (Figure 2a,b), showed XRD crystalline features similar to those of the precursors, but with low intensity in the case of nHAP. The positions of the diffraction peaks corresponding to the EMs (Figure 2a,b) were approximately the same as those of the precursors PCL, nHAP and MET (Figure 1 and Figure 2). These XRD patterns of the EMs (Figure 1 and Figure 2) indicated that there was no formation of a new crystal phase or the new changes in the crystal phases were too small to create a high signal.

**Figure 3:**
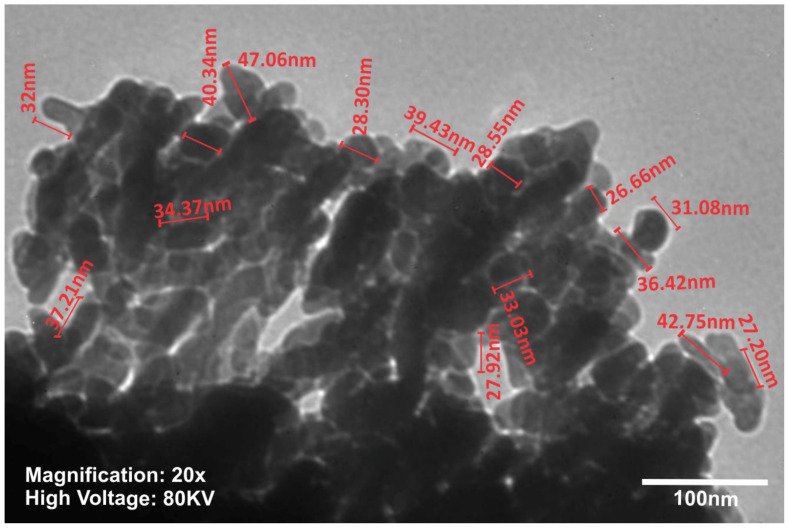
TEM image of the synthesized nHAP powder.

**Figure 4:**
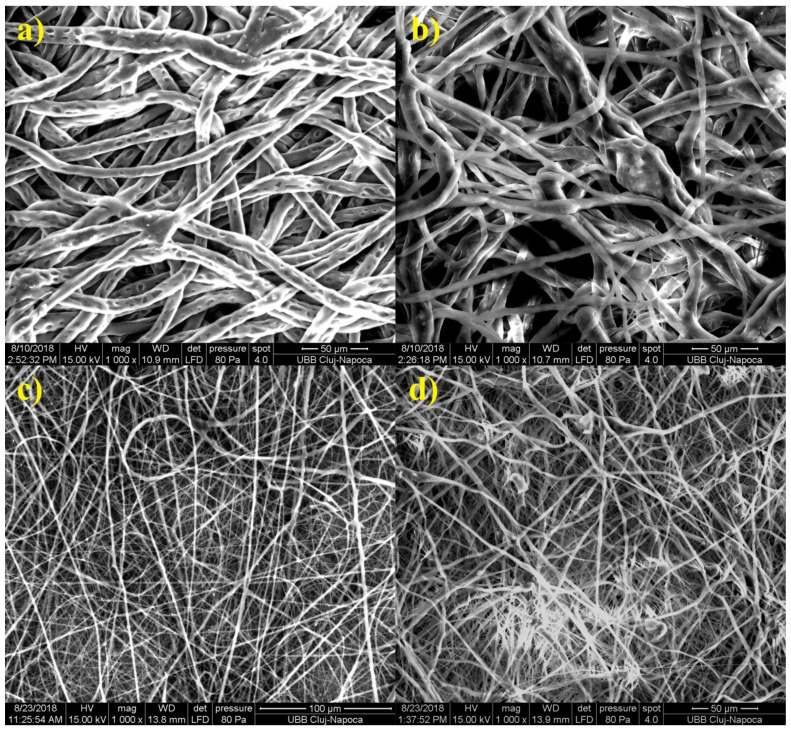
SEM micrographs of electrospun membranes: (**a**) PCL, (**b**) PCL-5% nHAP, (**c**) PCL-20% MET and (**d**) PCL-5% nHAP-20% MET.

**Figure 5:**
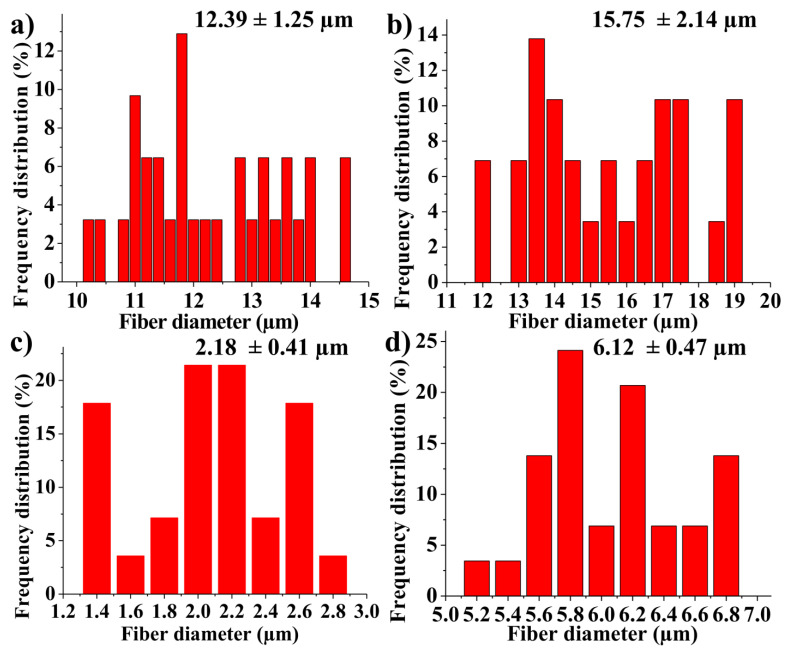
Fiber diameter frequency distributions from SEM images: (**a**) PCL, (**b**) PCL-5% nHAP, (**c**) PCL-20% MET and (**d**) PCL-5% nHAP-20% MET.

TEM investigation (Figure 3) of crystals of nHAP rod particles showed a mean length of 34.16 nm (±6.24 SD). The SEM images (Figure 4a–d) of the EMs (a) PCL, (b) PCL-5% nHAP, (c) PCL-20% MET and (d) PCL-5% nHAP-20% MET showed randomly oriented bead-free fibers, building a porous structure with interconnected macropores. The frequency distributions mean of fiber diameter (Figure 5a–d) of (a) PCL, (b) PCL-5% nHAP, (c) PCL-20% MET and (d) PCL-5% nHAP-20% MET showed values between 2.18 and 15.75 µm. From the SEM images, we could notice that the addition of nHAP filler increased the fiber diameter, and the highest diameter was registered for the PCL-5% nHAP membrane (Figure 4b and Figure 5b). The addition of MET to PCL decreased the fiber diameter (Figure 4c and Figure 5c) but the diameter increased if nHAP was added to the mixture of MET and PCL. The difference could be explained by the higher solubility of MET in the solvent than nHAP, which is a filler (Figure 4d and Figure 5d). The topography of the obtained EMs could be connected to a strong polymer–solvent interaction, solution viscosity or electrical conductivity and would influence the diameter, crystallinity, tensile strength and surface morphology of the electrospun fibers [22].

**Figure 6:**
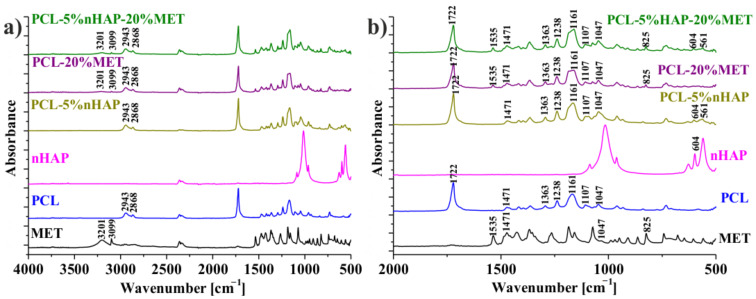
FTIR spectra of nHAP, MET, PCL, PCL-5% nHAP, PCL-20% MET and PCL-5% nHAP-20% MET: (**a**) FTIR spectrum detail 4000–500 cm^−1^ domain/interval; (**b**) FTIR spectrum detail 2000–500 cm^−1^ domain/interval (for a better visualization).

The FTIR spectra of the different precursors (nHAP, MET and PCL) and EMs (Table 1) are shown in Figure 6a,b. Figure 6b, which details the wavelength range between 2000 and 500 cm^−1^, shows the positions of the absorbance peaks more clearly. Thus, Figure 6a,b reveals MET characteristics like OH (alcohol) stretching band which appears at 3201 cm^−1^, C–H (aromatic) stretching at 3099 cm^−1^, C–NO_2_ and N–O stretching at 1535 cm^−1^, CH_2_ scissoring at 1471 cm^−1^, C–OH and C–O stretching at 1047 cm^−1^ and C–NO_2_ and C–N stretching at 825 cm^−1^ [23,24,25]. In Figure 6b, the spectrum of PCL-5% nHAP-20% MET and the specific bands of MET can be highlighted more easily. Values of the absorption bands of MET, such as N–O stretching at 1535 cm^−1^ and C–N stretching at 825 cm^−1^, did not show any displacements after it was introduced in the polymer (PCL) membrane. It can be said that no chemical bond was formed between drug and the polymer (PCL) matrix. Some characteristic peaks for MET, such as CH_2_ at 1471 cm^−1^ and C–OH and C–O at 1047 cm^−1^, were found to overlap with the PCL peaks in the drug-containing EMs.

**Figure 7:**
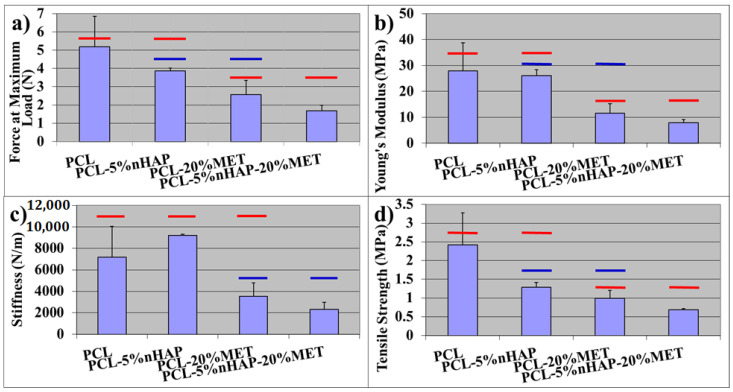
Mechanical properties of EMs: (**a**) force at maximum load (N) of EMs, (**b**) Young’s modulus (MPa) of EMs, (**c**) stiffness (N/m) of EMs and (**d**) tensile strength (MPa) of EMs. Red and blue horizontal bars indicate the mean values not statistically significantly different from each other, compared using Tukey’s test (*p* > 0.05).

The results of mechanical properties showed statistically significant differences (*p* < 0.05) when evaluated with one-way ANOVA statistical analysis. The highest values for mechanical properties (the force at maximum load, Young’s modulus and the tensile strength) (Figure 7a–d) were registered for the PCL membranes. These properties decreased in the following order: PCL > PCL-5% nHAP > PCL-20% MET > PCL-5% nHAP-20% MET. An explanation for this behavior could be the missing chemical bond between PCL, nHAP and MET, because the precursors MET and nHAP were embedded mechanically in the PCL matrix. The decrease in mechanical properties after the addition of nHAP is in agreement with other studies [18]. The results registered for all mechanical properties for PCL-5% nHAP show no statistical differences when compared with the PCL experimental EM (*p* > 0.5). There was one exception: the stiffness increased when 5 wt.% nHAP was added to the PCL matrix (*p* > 0.5). The addition of MET or 5% nHAP-20% MET to PCL decreased the stiffness.

The addition of MET to the PCL membrane caused a greater reduction in mechanical properties than the addition of nHAP. This could be explained by the difference in mechanical properties between nHAP and MET. When nHAP and MET were added together, the new EMs showed the lowest mechanical properties (Figure 7). All mechanical properties of PCL-20% MET and PCL-5% nHAP-20% MET showed statistically significant differences (*p* < 0.5) when compared with the PCL membrane. This behavior could be explained by the decrease in PCL percentage, because this is the only compound that contributed to the curing and polymer matrix construction. The stiffness (or rigidity) is the resistance of an elastic body to deflection or deformation by an applied force. Young’s modulus is a property of a material that indicates the ability to resist mechanical deformation [26]. The increase in stiffness with the addition of the filler is in agreement with other studies [27]. Caballé-Serrano et al. [28] investigated the mechanical properties of 15 commercial membranes and obtained the following results: Young’s modulus between 0.2 and 4.8 MPa; tensile strength between 0.1 and 1.8 MPa. If the membranes were tested after 2 and 4 min of hydration in distilled water, the results for the mechanical properties decreased [28]. When nHAP or MET was added, our obtained EMs showed lower mechanical properties than the PCL membrane but similar properties to those of commercial materials [28]. From the clinical point of view, more *in vitro* and *in vivo* tests are required in the future in order to see the advantages of nHAP or MET addition.

## 4. Conclusions

By using two dispersing agents, namely Darvan 821A and PVA, we have confirmed advantages in obtaining new nHAP particles. The imaging and characterization of nHAP particles by TEM, XRD and FTIR confirmed the presence of nHAP (mean diameter of 34 nm) obtained by new synthesis. XRD and FTIR showed the presence of PCL, nHAP and MET in newly obtained EMs without chemical bonds between them. The investigated mechanical properties (force at maximum load, Young’s modulus and tensile strength) decreased with the addition of nHAP and MET. The stiffness increased with the addition of 5 wt.% nHAP. The lowest mechanical properties were registered for the EM with nHAP and MET. The SEM images confirmed that all EMs showed a fibrous and porous structure. The greatest diameter of the fiber was obtained when 5 wt.% nHAP was added, and the diameter decreased when the filler was replaced with MET. Therefore, all null hypotheses for the newly obtained EMs are rejected. New experimental EMs with nHAP and MET could be promising materials for guided bone regeneration or other tissue engineering applications. These results encourage us to pursue further investigation *in vitro* and *in vivo* for the clinical application of these EMs.

## Figures and Tables

**Table 1:** The composition of experimental electrospun membranes (EMs).

No.	Code	Composition of Experimental EM		
		PCL (wt.%)	nHAP (wt.%)	MET (wt.%)
1	PCL	100	0	0
2	PCL-5% nHAP	95	5	0
3	PCL-20% MET	80	0	20
4	PCL-5% nHAP-20% MET	75	5	20

## Data Availability

No new data were created or analyzed in this study. Data sharing is not applicable to this article.
